# Investigation of the Interaction Between Angiotensin-Converting Enzyme (ACE) and ACE-Inhibitory Tripeptide from Casein

**DOI:** 10.3390/ijms252313021

**Published:** 2024-12-04

**Authors:** Cuicui Yang, Tianzhao Xie, Mengmeng Cai, Xiaoting Xu, Muzijun Li, Pengru Liu, Xiongdiao Lan

**Affiliations:** Guangxi Key Laboratory for Polysaccharide Materials and Modifications, Guangxi Higher Education Institutes Key Laboratory for New Chemical and Biological Transformation Process Technology, School of Chemistry and Chemical Engineering, Guangxi Minzu University, Nanning 530006, China; yangcucu@outlook.com (C.Y.); 19127621550@163.com (T.X.); a13317177396@163.com (M.C.); xiaoting5408@163.com (X.X.); 18376523197@163.com (M.L.)

**Keywords:** angiotensin-converting enzyme inhibitory peptides, casein, stability, molecular docking, spectroscopy

## Abstract

Angiotensin-converting enzyme (ACE) inhibitory peptides exhibit antihypertensive effects by inhibiting ACE activity, and the study of the interaction between ACEs and inhibitory peptides is important for exploring new therapeutic strategies. In this study, the ACE-inhibitory peptide isolated from casein hydrolysate with the amino acid sequence Leu–Leu–Tyr (LLY) exhibited high ACE-inhibitory activity and stability, which holds significant implications for biochemistry and pharmaceutical applications. Furthermore, systematic investigations were conducted on the interaction between ACE and LLY through various approaches. The Lineweaver–Burk plot indicated the non-competitive inhibition pattern of LLY, suggesting that it binds to the enzyme at the non-active site, and the results were further validated by a molecular docking study. Additionally, multispectral experiments and atomic force microscopy were conducted to further elucidate the underlying mechanism of peptide activity. The findings indicated that LLY could induce a conformational change in ACE, thereby inhibiting its activity. This study contributes to a deeper understanding of the mechanism of action of ACE-inhibitory peptides and bears important significance for drug development in hypertension.

## 1. Introduction

Hypertension is a significant chronic condition that elevates the risk of mortality from cardiovascular, cerebrovascular, and renal diseases and stands as one of the primary contributors to global mortality. According to the World Health Organization, the prevalence of high blood pressure among individuals aged 50–79 is estimated at 49% worldwide, indicating that nearly one in three adults globally suffers from hypertension [1].

Angiotensin-converting enzyme (ACE), also referred to as kallikrein II or peptidyl-prolyl dipeptidase, plays a vital role in the regulation of blood pressure and electrolyte homeostasis through the renin–angiotensin–aldosterone system (RAAS) and the kallikrein–kinin system (KKS) [2,3]. In RAAS, ACE converts angiotensin I to angiotensin II, which has a strong vasoconstriction effect and stimulates the release of aldosterone from the adrenal cortex, all of which increase blood pressure [4,5]. In KKS, ACE causes changes in the molecular structure of bradykinin through the cleavage of the C-terminal dipeptide, resulting in a loss of activity, which leads to vasoconstriction and increased blood pressure [6,7]. The inhibition of ACE activity represents one of the most effective strategies for managing elevated blood pressure. Consequently, ACE inhibitors are extensively utilized in the treatment of cardiovascular diseases and rank among the most commonly prescribed antihypertensive medications [8,9]. ACE belongs to a widely distributed class of enzymes (dipeptidyl peptidases), so its inhibitors can be found in various plants and animals [4]. Furthermore, ACE inhibitors derived from natural sources are devoid of side effects and are widely regarded as safe [10]. In recent years, numerous researchers have successfully isolated and identified ACE-inhibitory peptides from various natural animal and plant protein sources, including rapeseed protein [11], soy protein [12], fish protein [13], walnut [14], jujube [15], earthworm protein [16], and so on.

Casein, abundant in milk, is a rich source of ACE-inhibitory peptides due to its high amino acid content [17]. Jiang et al. [18] isolated a novel ACE-inhibitory peptide from bovine casein hydrolyzed by the AS1.398 neutral protease. The amino acid sequences identified were Arg–Tyr–Pro–Ser–Tyr–Gly (J-casein; f25-30) and Asp–Glu–Arg–Phe (J-casein; f15-18), respectively. These peptides demonstrated significant antihypertensive activity following oral administration in spontaneously hypertensive rats. In another study, Cadée et al. [19] investigated a casein-derived protein hydrolysate (C12 peptide), demonstrating the ability to reduce blood pressure in individuals with prehypertension. Additionally, Tu et al. [20] identified a new ACE-inhibitory heptapeptide EKVNELSK from alpha s19 casein (fragment 35–42), indicating vigorous ACEI activity. These findings suggest that casein is an excellent source of ACE-inhibitory peptides and that peptides extracted from caseins may be beneficial components of antihypertensive functional foods or drugs.

In our previous study, a novel ACE-inhibitory peptide, identified as LLYQEPVLGPVR and exhibiting moderate ACE-inhibitory activity with an IC_50_ value of 273.5 ± 5.4 μmol·L^−1^, was obtained from casein through enzymatic hydrolysis using trypsin and pepsin [21]. However, LLYQEPVLGPVR was digested after simulated gastrointestinal digestion and produced shorter peptides, which enhanced ACE-inhibitory activity. The produced tripeptide Leu–Leu–Tyr (LLY) displays an impressive IC_50_ value of 44.16 ± 2.45 μmol·L^−1^, and nonapeptide Gln–Glu–Pro–Val–Leu–Gly–Pro–Val–Arg (QEPVLGPVR) shows a less-potent inhibition, with an IC_50_ value of 18.72 ± 0.78 mmol·L^−1^. The comparative evaluation revealed that tripeptide LLY exhibited superior inhibitory activity, suggesting its potential for further exploration. Relevant studies have demonstrated that small peptides (fewer than three amino acids) exhibit greater efficacy than larger ones [22]. Reducing the peptide length from ten amino acids to four or even two can significantly enhance its stability and absorption efficiency in vivo [23]. Short peptides offer numerous advantages over their larger counterparts, including enhanced selectivity and specificity, biodegradability and biocompatibility, high safety profiles, low toxicity (attributable to their safe metabolite, amino acids, and limited potential for accumulation in the body), and reduced immunogenicity [24]. Tripeptide LLY represents a short peptide that may exhibit low immunogenicity, thereby increasing the prospects for its successful application beyond the intestinal environment. Peptides comprising two or three amino acid residues have been reported to effectively traverse the digestive epithelial barrier and reach blood vessels, facilitating access to peripheral organs and exerting beneficial effects on the organism [22,25]. Moreover, several laboratories have recently identified that short peptides consisting of two or three amino acids are well absorbed in the mammalian gut via the peptide transport system [26]. Richard et al. [27] discovered that specific highly potent ACE-inhibiting peptides found in fermented milk demonstrate resistance to gastrointestinal digestion; these peptides successfully cross mucosal barriers and withstand further degradation by serum peptidases while mediating physiological responses. Consequently, as a potential oral therapeutic agent, tripeptide LLY holds significant prospects and essential research value.

Early studies on ACE-inhibitory peptides primarily focused on their preparation, purification, and amino acid sequence analysis. However, direct investigations into the interaction between ACE inhibitors and ACE, as well as the underlying mechanisms, are still scarce. Therefore, this study aims to elucidate the structure and molecular mechanisms of peptide compounds obtained from natural sources to introduce new inhibitors to control blood pressure. In this paper, the casein-derived tripeptide LLY was selected as the research object. Molecular docking of tripeptide LLY and ACE was carried out, and a spectral analysis, including ultraviolet (UV), fluorescence, and circular dichroism (CD) spectrum, was performed to explore the interaction mechanism between LLY and ACE. Furthermore, atomic force microscopy (AFM) was used to observe the structural changes caused by the peptide binding with ACE. The findings of this study confirm LLY’s inhibitory effect on ACE and offer new insights into its potential as a natural antihypertensive agent.

## 2. Results

### 2.1. Evaluation of Half Maximal Concentration—IC_50_

**Figure 1:**
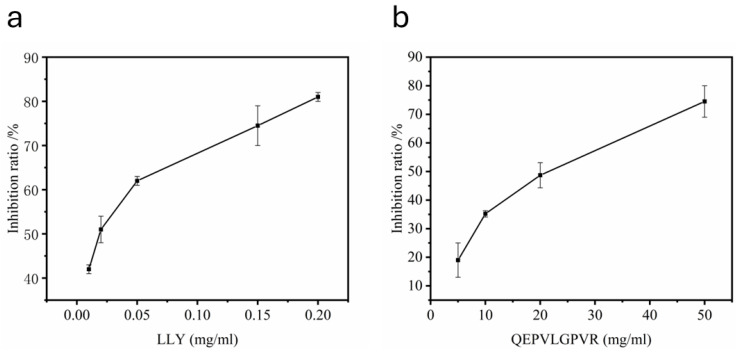
The ACE inhibitor ratio at different concentrations of ACE-inhibitory peptides. (**a**) LLY and (**b**) QEPVLGPVR.

The IC_50_ value was defined as the concentration of peptide required for binding to the ACE and inhibiting ACE activity by 50% [28]. The concentration of ACE-inhibitory LLY was taken as the horizontal coordinate. The inhibition rate of ACE was taken as the vertical coordinate. The inhibition rates of the different concentrations of ACE-inhibitory peptides are shown in Figure 1, and the IC_50_ value was calculated by curve fitting. The IC_50_ value of tripeptide LLY was estimated to be 44.16 ± 2.45 μmol·L^−1^ (Figure 1a), and the IC_50_ value of QEPVLGPVR was calculated to be 18.72 ± 0.78 mmol·L^−1^ (Figure 1b). The IC_50_ value of tripeptide LLY is significantly lower than that of the nonapeptide QEPVLGPVR, indicating the inhibitory capacity of LLY is more potent than that of the LLYQEPVLGPVR.

### 2.2. Stability Analysis

**Figure 2:**
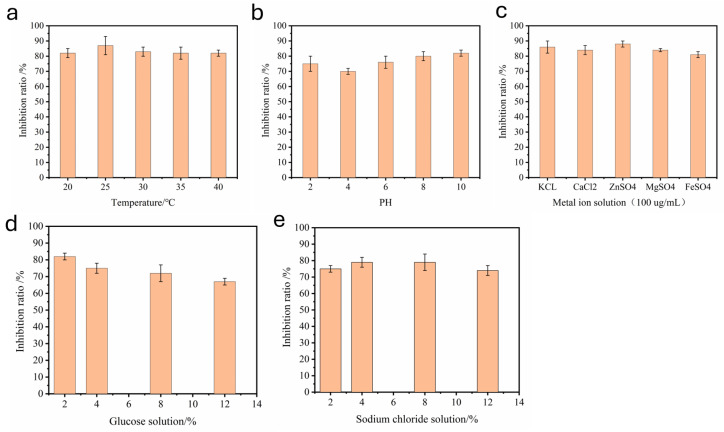
Stability analysis of ACE-inhibitory peptide LLY. (**a**) Thermal stability; (**b**) pH stability; (**c**) different metal ion stability; (**d**) stability at different glucose concentrations; (**e**) stability at different salt concentrations. Different superscripts note the significant differences (*p* < 0.05). All results are average ± SD from three determinations.

The stability tests of ACE-inhibitory peptide LLY under different environmental conditions are shown in Figure 2 below. All fractions showed a significant difference (*p* < 0.05) in ACE-inhibitory activity. Figure 2a shows that the ACE-inhibitory peptide LLY is relatively stable at 20 to 40 °C. When the temperature was 25 °C, the inhibition rate of LLY to ACE reached the maximum (87%). While the temperature exceeded 25 °C, the inhibition rate of LLY to ACE began to decline, but it still had more than 80% activity after treatment at 40 °C. Thus, the ACE-inhibitory peptide LLY has good thermal stability and can maintain high activity under thermal environment conditions. The pH stability of the ACE-inhibiting peptide LLY is illustrated in Figure 2b. The result demonstrates that, as the pH value increases, the inhibition of ACE by LLY initially decreases and then increases. When the pH value is 4.0, LLY exhibits the lowest degree of inhibition on ACE, with an inhibition rate still at 70%. This indicates that LLY demonstrates good stability across varying pH levels.

The effect of metal ions on the ACE-inhibitory activity of LLY is shown in Figure 2c. The results indicated that adding K^+^, Ca^2+^, Mg^2+^, and Fe^2+^ ions had little effect on the LLY inhibition rate [29]. The metal ion Fe^2+^ has a higher inhibition degree than other metal ions, and the addition of Fe^2+^ can still have an inhibition rate of more than 81%. So, it is speculated that the ACE-inhibitory peptide LLY has good metal ion stability. The effects of adding different glucose concentrations on the ACE-inhibitory activity of LLY are shown in Figure 2d. It can be seen that, with the increase in the glucose concentration, the inhibition rate of LLY on ACE continues to decrease, and the inhibition rate of LLY is 82% when the glucose concentration is 2%. However, when the glucose concentration reached 12%, the inhibition rate of ACE decreased to 67%, indicating that the increase in glucose concentration had a more significant effect on the inhibition rate of ACE-inhibitory peptides. The impacts of different salt concentrations on the ACE-inhibitory activity of LLY are shown in Figure 2e. With the increase in salt concentration, the inhibition of LLY on ACE first increased and then decreased. When the salt concentration was 12%, the inhibition rate dropped to 74%. This change might result from destroying amino acid side-chain groups in specific peptide structures caused by high NaCl content.

### 2.3. Inhibitory Pattern of LLY on ACE

**Figure 3:**
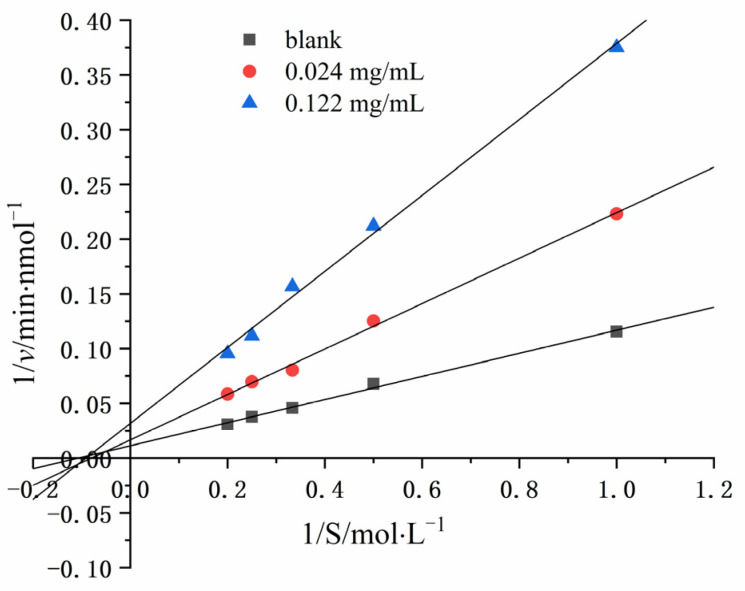
Lineweaver−Burk plots of the reactions of ACE in the presence of LLY.

The inhibition pattern of LLY on ACE was studied using the Lineweaver–Burk figure, as shown in Figure 3. With the rise in the LLY concentration, the slope of the line segment and the intercept increase, and the three lines converge at one point on the x-axis. That is, the K_m_ value remains unchanged, with K_m_ being 10.82 mol·L^−1^. V_m_ decreases as the concentration of the inhibitory peptide increases, indicating that LLY pertains to non-competitive inhibition, and it was bound to the enzyme at the non-active site.

### 2.4. Molecular Docking Analysis

**Figure 4:**
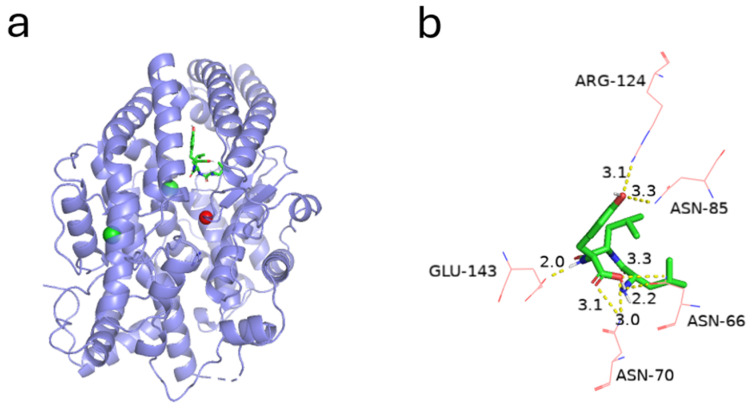
Docking simulation of LLY binding to ACE. (**a**) Docking simulation of LLY (green) binding to ACE (purple). A zinc ion (red) was present in the active site of ACE; (**b**) interaction between LLY (shown as green sticks) and the residues of ACE (shown as red lines) is shown.

Molecular docking of LLY with ACE was carried out using Auto Dock Vina (1.1.2) to predict the ligand−receptor interaction mechanism, as shown in Figure 4. The extracted ligand Lisinopril was re-docked to the ACE protein to validate the molecular docking methodology. The root-mean-square deviation (RMSD) between the conformation calculated by PyMol and the original ligand conformation is 1.329 Å, which falls well within the 2 Å grid spacing utilized in the docking process [30]. It shows that the docking method adopted is effective and reliable. The pose with the lowest binding energy was recognized as the best conformation for further analysis. According to the docking results, the lowest energy of the most stable structure combining LLY and ACE is −7.2 kcal·mol^−1^, as shown in Figure 4a. The hydrogen bond is considered the most crucial factor influencing the binding stability. Figure 4b illustrates the formation of seven hydrogen bonds between the ACE-inhibitory peptide LLY and five amino acid residues of ACE, namely Glu-143 (2 Å), Asn-66 (2.2 and 3.3 Å), Asn-70 (3 and 3.1 Å), Asn-85 (3.3 Å), and Arg-124 (3.1 Å). None of these amino acids are located in the active site of ACE. Additionally, Trp-357, Leu-139, and Glu-143 form hydrophobic interactions with LLY molecules within ACE’s structure. The results from molecular docking and Lineweaver–Burk inhibition mode analysis demonstrated that the binding inhibition mode of LLY and ACE is non-competitive.

### 2.5. UV Spectral Analysis

**Figure 5:**
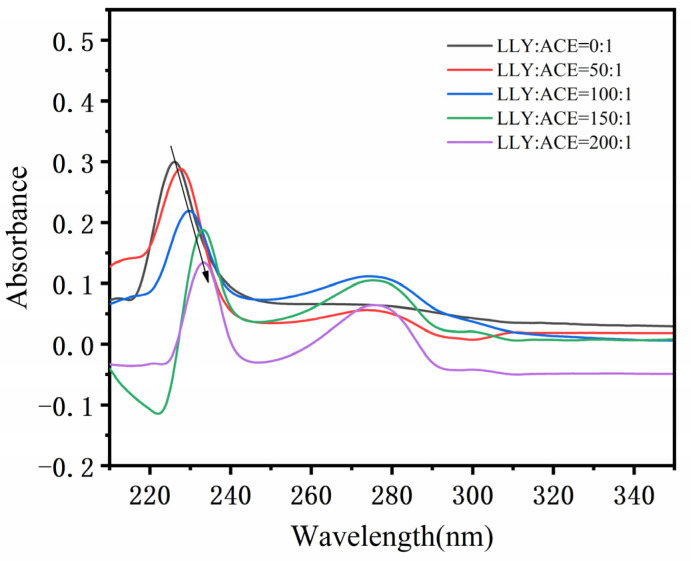
The UV spectra of ACE-inhibitory peptide LLY binding to ACE.

Figure 5 shows the UV spectra of different proportions of the inhibitory peptide LLY interacting with ACE. As can be seen from Figure 5, When the ACE-inhibitory peptide LLY was added, the absorbed light intensity at 224 nm gradually decreased with the increase in the inhibitory peptide concentration (subtractive effect), indicating that the addition of LLY caused the change in ACE conformation (peptide chain), and the energy of its electronic transition decreased (π → π* transition), resulting in a subtractive effect. Moreover, the maximum absorption wavelength at 224 nm showed a redshift (224 nm → 233 nm) with the increase in the ACE-inhibitory peptide concentration, indicating that the addition of LLY changed the hydrophobicity of the ACE peptide chain and improved the stretch of the peptide chain. Before and after the addition of LLY, the absorption peak at 280 nm also changed, but not regularly, which indicates that the addition of LLY will also affect the aromatic amino acids, such as Tyr of ACE, and then affect the change in the ACE structure.

### 2.6. Fluorescence Spectral Analysis

#### 2.6.1. Fluorescence Spectrum

**Figure 6:**
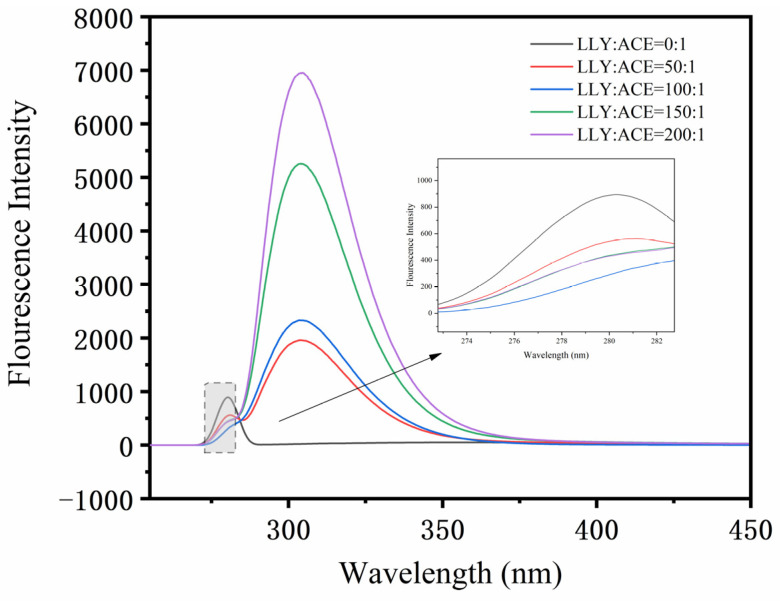
Fluorescence spectra of ACE-inhibitory peptide LLY binding to ACE.

The fluorescence spectra of the interaction between inhibitory peptide LLY and ACE are shown in Figure 6. In the 4-(2-hydroxyethyl)-1-piperazineethanesulfonic acid (HEPES) system, the maximum emission peak of ACE is about 291 nm. After the addition of LLY, the fluorescence intensity of the maximum emission peak of ACE decreases, and its position shifts. The result illustrates that, as the concentration of LLY increases, there is a gradual decrease in fluorescence intensity. However, after saturation occurs following combination with ACE, irregularities are observed in the fluorescence quenching process. It can be inferred that this process may result from multiple pathways acting comprehensively on LLY’s quenching effect on ACE [31,32].

#### 2.6.2. The Three-Dimensional Fluorescence Spectra

**Figure 7:**
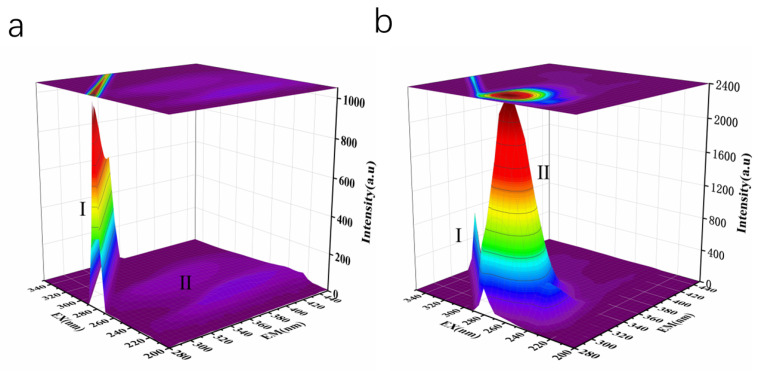
The 3D fluorescence spectra of ACE-inhibitory peptide LLY interacting with ACE. (**a**) ACE; (**b**) ACE + LLY.

Three-dimensional (3D) fluorescence technology was used to study the interaction between LLY and ACE. As shown in Figure 7, peak I is the Raleigh scattering peak, with its emission wavelength equal to the excitation wavelength [33]. The gradual decrease in the fluorescence intensity of the peak after the addition of LLY indicates the formation of the ACE–LLY complex and its impact on the microenvironment of Trp and Tyr residues. From Table 1, it can be observed that peak II (λex/λem = 275/305 nm) primarily reflects the fluorescence spectral behavior of the LLY peptide backbone. Adding LLY enhances peak II’s value (λex/λem = 275/305 nm), indicating an increased ACE molecule diameter due to LLY addition. Therefore, the fluorescence spectra and the 3D fluorescence spectra showed that the inhibitory peptide LLY interacts with ACE to form an ACE–LLY complex. The interaction between the two causes a change in ACE conformation, resulting in LLY’s inhibition of ACE activity.

### 2.7. CD Chromatographic Analysis

**Figure 8:**
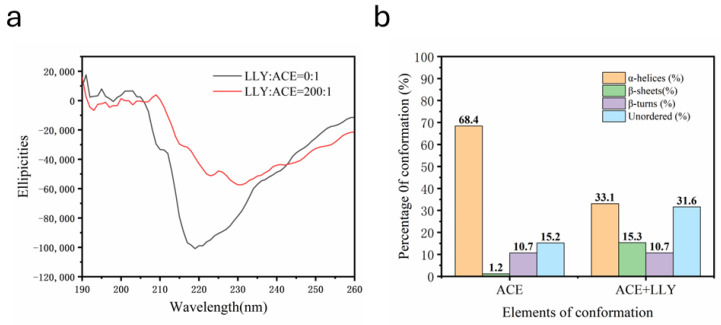
The secondary structure of ACE affected by LLY. (**a**) CD spectrum; (**b**) percentage of ACE secondary structures in the absence and presence of LLY.

Figure 8 below shows the secondary structure diagram of ACE in the HEPES buffer solution system before and after the addition of inhibitory peptide LLY analyzed by CD. As shown in Figure 8a, the combination with LLY significantly affects the CD spectrum of ACE, and the peak intensity at 219 nm decreases with redshift (219 nm → 233 nm). This redshift is possibly attributed to the elongation of ACE upon the addition of LLY, resulting in a partial loss of its α-helical structure. As depicted in Figure 8b, the introduction of the ACE-inhibitory peptide LLY resulted in a reduction of the α-helix content of ACE from 68.4% to 33.1%, accompanied by an increase in the random coil content from 15.2% to 31.6% and the β-sheet content from 1.2% to 15.3%. This suggests that the α-helical structure of ACE undergoes a gradual transition towards a random coiled and β-folded conformation upon interaction with LLY. Therefore, adding LLY induces a reorganization of the ACE peptide chain, leading to alterations in its original structure and spatial conformation and resulting in decreased ACE activity.

### 2.8. AFM Analysis

**Figure 9:**
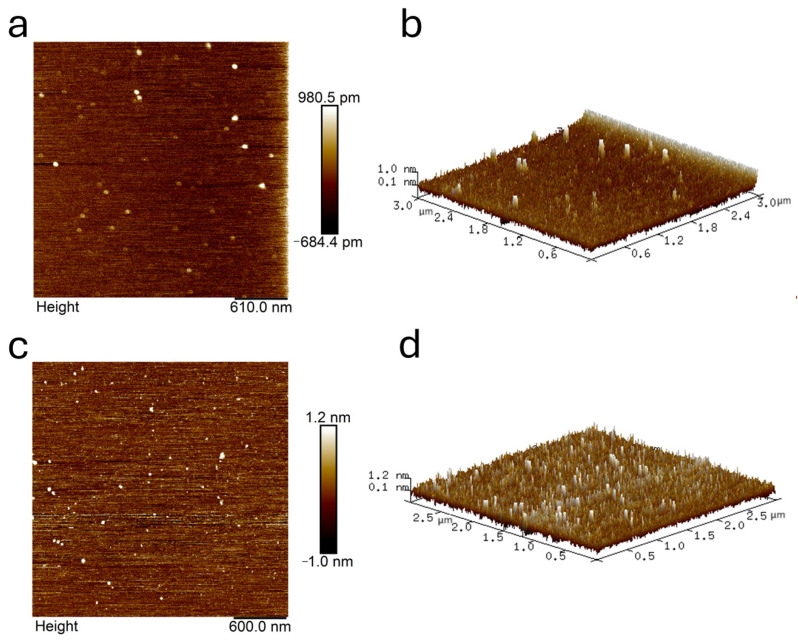
AFM results of ACE-inhibitory peptide LLY interacting with ACE. (**a**) AFM-2D image of ACE; (**b**) AFM-3D image of ACE; (**c**) AFM-2D image of LLY–ACE; (**d**) AFM-3D image of LLY–ACE.

The fine morphological changes in the 2D and 3D shapes of ACE and the addition of LLY peptide were characterized using atomic force microscopy, as shown in Figure 9. The particle analysis feature in the Nanoscope Analysis 1.5 software (Bruker, Beijing, China) was utilized to evaluate the height and diameter of both types of particles. As illustrated in Figure 9a,b, ACE particles exhibit a dispersed distribution. The particle analysis function determined that their average diameter and height are 53.31 nm and 2.80 nm, respectively, indicating relatively large sizes. In contrast, following the introduction of the LLY peptide, as depicted in Figure 9c,d, the distribution appears more concentrated, with a notable increase in the total particle count. The average measurements for this scenario were found to be 40.28 nm for the diameter and 3.0 nm for the height, reflecting smaller molecular dimensions. The result shows that the interaction between ACE and the LLY modifies the spatial structure of the ACE peptide chain, ultimately resulting in diminished ACE activity.

## 3. Discussion

The ACE-inhibitory activity of peptides is intimately correlated with their structural characteristics. Among the numerous reported peptide sequences, effective ACE inhibitor peptides are typically short, with a length of 2 to 12 amino acids [34]. It has been reported that the presence of repeated amino acids in the peptide sequence of tripeptides enhances their biological activity compared to those containing distinct single amino acids. Observations indicate that polypeptide sequences featuring repeated amino acids, such as Ala–Ala, Pro–Pro, and Leu–Leu, confer more potent inhibitory activity to tripeptides [3]. Jiang et al. [35] discovered that many short-chain peptides characterized by hydrophobicity and other amino acids, such as proline (Pro), tyrosine (Tyr), phenylalanine (Phe), or tryptophan (Trp) at the C-terminal, are regarded as effective ACE-inhibitory peptides. Kheeree et al. [36] included hydrophobic aromatic amino acids like Phe and Trp at the C-terminal that significantly influence the binding affinity with ACE. Furthermore, Yin et al. [37] demonstrated that Pro and Phe enhance the binding capability between peptides and ACE. In this study, we focus on the tripeptide LLY derived from casein, which contains a repetitive amino acid sequence of Leu–Leu. Additionally, its C-terminal features a hydrophobic amino acid Tyr. Its specific amino acid sequence and the structural attributes of this tripeptide are likely pivotal to its high efficacy in ACE inhibition.

The thermal stability and pH stability of peptides are significant factors to consider in the production and processing of these compounds [38]. Food-derived inhibitory peptides have the potential to be incorporated into foods as ingredients, which requires them to demonstrate stability in various food systems and processing procedures. Factors such as the addition of salt, sugar, and water, or the use of metal containers during food processing can influence the properties and biological activities of polypeptides [39]. In this study, LLY was evaluated for its thermal stability, pH stability, and resilience after the addition of varying concentrations of metal ions, salt, and sugar. The results indicated that LLY exhibited good stability under these conditions, suggesting its potential application in future food-processing endeavors.

The peptide inhibition of enzymes can be classified into three types, namely competitive inhibition, non-competitive inhibition, and mixed inhibition [40]. Competitive inhibitors reversibly contend with the substrate for binding to the ACE active site. On the contrary, non-competitive inhibitors do not attach to the ACE active site but reversibly bind to other sites. Mixed inhibition involves binding to both the active and non-active sites of the enzyme [41]. In the Lineweaver–Burk diagram, if the sample curve intersects the X-axis of the blank sample, the active peptide is classified as a non-competitive inhibitor. If it intersects the Y-axis, it indicates competitive inhibition. Conversely, if it does not intersect either axis, it is categorized as a mixed inhibitor. The Lineweaver–Burk plot for LLY (Figure 3), where three straight lines converge at a point on the x-axis, suggests that LLY acts as a non-competitive inhibitor of ACE. Non-competitive inhibitors usually have reversible binding properties and do not affect the substrate binding process because they can act on different non-active sites, inducing conformational changes that affect enzyme activity [42]. This indicates that LLY binds to the non-active site of ACE, without affecting the substrate binding process to exert its inhibitory ability. Peptides from some food sources have also been reported in non-competitive inhibition modes, such as mushrooms [43], oysters [44], etc.

Molecular docking can further understand the molecular mechanism of the interaction between ACE and ACE [45]. ACE contains three active site pockets, namely S1 (Ala354, Glu384, and Tyr523), S2 (Gln281, His353, Lys511, His513, and Tyr520), and S1’ (Glue162) [46]. Molecular docking studies indicate that LLY does not interact with the active site of ACE, suggesting that LLY functions as a non-competitive inhibitor of ACE. This finding is consistent with the results from inhibitory mechanism investigations. Hydrogen bond interactions have been demonstrated to play a crucial role in stabilizing the docking ligand complexes [20]. Fu et al. [47] reported that there were multiple hydrogen bonds between peptides and ACE, indicating that peptides inhibited ACE activity through hydrogen bonding to stabilize enzyme-peptide complexes. The inhibitory effect of LLY peptides may be attributed to their capacity to form multiple hydrogen bonds with ACE. Previous studies have extensively discussed this issue. For example, Zaharuddin et al. [48] investigated six inhibitory peptides derived from the seed protein of red flax and identified that LYWSYLYN exhibited the strongest binding affinity and inhibitory effect on ACE, forming six hydrogen bonds with residues Asp377, His353, Glu162, His513, Tyr523, and Glu143. In this study, the LLY inhibitory peptide also established hydrogen bonds with Glu143. In addition, the hydrogen bond distance between the inhibitory peptide and the amino acid residues was short, indicating that the peptide had a strong binding affinity with ACE [49,50]. The hydrogen bond distance between LLY and the ACE amino acid residue is less than 3.5 Å (Figure 4b), indicating that LLY has a strong binding affinity to ACE and enhances its inhibitory activity.

It is well known that UV, the fluorescence spectrum, and CD chromatography are effective methods to explore the structural changes and intermolecular interactions of proteins [51,52]. As shown in the UV spectrogram (Figure 5), there are two absorption peaks at 224 nm and 280 nm. A strong absorption peak at 224 nm is characteristic of the peptide skeleton, while a weak absorption peak at 280 nm is typical of aromatic amino acids [31]. The peak intensity at 224 nm showed a significant redshift, indicating that the ACE skeleton was disturbed [32]. Guo et al. [51] discovered that, in the UV and fluorescence spectra, the addition of starch decreased the ultraviolet absorption intensity and fluorescence intensity of whey protein isolate (WPI). The results indicated the formation of the starch–WPI complex, and the amount of the starch–WPI complex was positively correlated with the starch concentration. Memarpoor-Yazdi et al. [15] utilized CD spectroscopy and found that the α-helix content of ACE decreased and the content of random coil increased after the interaction with the inhibitory peptide. These changes indicated mild denaturation and the loss of enzyme activity. That is, a significant change occurred in the ACE conformation. Guo et al.’s experiment provided compelling evidence supporting our experimental findings and confirmed that the interaction between ACE-inhibitory peptides and ACE resulted in enzyme inactivation. AFM can observe the 3D structures of various biological molecules, such as proteins and peptides, to study the surface structure and properties of the sample being tested [53,54]. AFM analysis showed an increase in the number of molecules and a decrease in particle size after the addition of LLY. This change may result from ACE interacting with functional groups on the LLY peptide to create an ACE-LLY complex, which alters the conformation of the peptide and leads to an increased number of molecules along with reduced particle size and enhanced concentration in distribution. This study is similar to previous research by Zeng et al. [55], who used atomic force microscopy (AFM) to study the average height of individual molecules of C23O-2G after the addition of catechol to fall below 0.1 nm, suggesting that the binding of catechol to C23O-2G leads to the tightening and contraction of the protein framework. Through AFM characterization analysis, it was further confirmed that the interaction between ACE-inhibitory peptides and ACE is likely attributed to complex formation, which induces a change in the spatial conformation of ACE. The experimental results are consistent with the properties of the non-competitive inhibitors described above.

## 4. Materials and Methods

### 4.1. Materials and Reagents

ACE of rabbit lung origin was purchased from Aldrich (Shanghai, China). Hippuryl–histidyl–leucine (HHL), methanol, and trifluoroacetic acid (TFA) were purchased from Sigma Chemical Co. (Saint Louis, MO, USA). The LLY (grade > 98%) was synthesized by GL Biochem Ltd. (Shanghai, China). All other reagents are analytically pure and can be used without further purification.

### 4.2. Determination of Half-Maximal Concentration IC_50_

The ACE and inhibitory activity were evaluated by measuring the amount of hippuric acid (HA) described by Chen et al. [56] with some modifications. Diverse concentrations of LLY solutions (0.01, 0.02, 0.05, 0.15, and 0.2 mg/mL) and an HHL solution at a concentration of 5 mmol·L^−1^ were formulated utilizing a boric acid (BBS) buffer solution with a concentration of 0.1 mol·L^−1^ (comprising 0.3 mol·L^−1^ NaCl; pH = 8.3). Thirty microliters of ACE solution were combined with 100 μL BBS buffer and 30 μL of variously concentrated LLY solutions. A control group devoid of peptide inhibitors was established. The sample was mixed well, preheated for 10 min in a 37 °C water bath shaker. After the water bath, add 40 μL HHL solution, mixed again, and reacted at 37 °C water bath shaker for 15 min. Finally, the reaction was stopped by adding 150 μL of 1.0 mol·L^−1^ HCl. The entire reaction was conducted in a water bath maintained at 37 [57]. The prepared samples were measured for the content of HA by high-performance liquid chromatography (HPLC). The HA production was determined by Agilent 1260 HPLC (Agilent, CA, USA). The chromatographic conditions were: column: Zorbax SB-C18 and detector: DAD diode array detector. The mobile phase was 15% methanol (containing 0.1% TEA) and 85% water (containing 0.1% TEA). The flow rate was 1 mL·min^−1^ and the detection wavelength was 228 nm. Each sample was measured three times, and the arithmetic mean of the three measured values was calculated as the representative value of the sample.

The inhibition percentage (IP) is computed as follows [37]:

Inhibition percentage (IP) = (A_1_ − A_2_)/A_1_ × 100%

where A_1_ denotes HA content in the uninhibited blank control group, and A_2_ signifies HA content in the samples treated with inhibitory peptides.

### 4.3. Stability Evaluation of ACE-Inhibitory Peptide LLY

The LLY solution at a concentration of 0.2 mg/mL was incubated in a water bath at temperatures of 20, 25, 30, 35, and 40 °C for 2 h to assess its in vitro inhibitory activity on ACE across varying temperatures. LLY was dissolved in BBS buffers (0.1 mol·L^−1^) at different pH values (2, 4, 6, 8 and 10), with a final concentration of 0.2 mg/mL. Incubated in a water bath at 37 °C for 2 h, and then the ACE-inhibitory activity of the peptide was measured with the peptide untreated as the comparison. The synthesized peptide solution (0.2 mg/mL) was mixed with different 100µg/mL metal ion solutions (KCl, CaCl_2_, ZnSO_4_, MgSO_4_, FeSO_4_), different concentrations (2%, 4%, 8%, 12%) of glucose solutions, and different concentrations (2%, 4%, 8%, 12%) of salt solutions. Incubate in 37 °C water bath for 2 h. Then the ace inhibitory activity of the peptide was determined by comparing it with the untreated peptide. The above inhibition rate was determined by the same method as Section 4.2. The effects of different environmental conditions on the inhibitory activity of LLY in vitro were determined. The untreated peptide solution served as the blank control group, ensuring the accuracy of our results.

### 4.4. ACE Inhibition Kinetics

The kinetics of inhibitory ACE of LLY were determined by the Lineweaver–Burk plots according to the reported method [50]. Solutions of HHL with concentrations of 1, 2, 3, 4, and 5 mmol·L^−1^ were prepared using a 0.1 mol·L^−1^ borate-buffered saline (0.3 mol·L^−1^ NaCl at pH 8.3). A total of 30 μL of ACE solution were taken and mixed with 100 μL BBS solution and 30 μL of ACE-inhibitory peptide LLY at different concentrations (10/50 mg/mL). After shaking, incubate in a 37.0 °C water bath for 10 min. After that, different concentrations of HHL solution 40 μL were added, mixed in shock, and reacted at 37 °C for 5 min. Finally, the reaction was terminated by adding 150 μL of 1.0 mol·L^−1^ HCl. After filtration through a cellulose filter membrane with a pore size of 0.22 μm, the HA content was determined using HPLC, and the formation rate of HA was calculated [32]. The method for determining the generated HA content is the same as described in Section 4.2 above. The inhibition pattern of LLY inhibitory peptide on ACE was then determined by a Lineweaver–Burk analysis based on the relationship between different substrate concentrations and the HA production rate [58]. The inhibition constant (K_i_) was the X-axis intercept of the curve. The Y-axis showed the slope of the Lineweaver–Burk line, and the X-axis indicated the peptide concentration [59].

### 4.5. Molecular Docking

The LLY structure was generated using ChemDraw21.0 (PerkinElmer, Waltham, MA, USA), with ChemDraw 3D 21.0 software (PerkinElmer, Waltham, MA, USA) utilized to map the LLY 3D structure and MM2 force fields employed for energy minimization. The crystal structure of ACE (PDB: 1O86) was obtained from the Protein Data Bank [46]. Lisinopril and water molecules were removed using Pymol 3.1 (Schrödinger, New Work, NY, USA)while the cofactors zinc ion and chlorine atom were retained. The receptor protein underwent hydrogenation, and charge balance adjustments were made using Auto Dock Tools 1.5.7 (Scripps Research, La Jolla, CA, USA). Molecular docking simulations were performed using Auto dock Vina 1.1.2 software (Scripps Research, La Jolla, CA, USA), and the best conformation of LLY and ACE was selected based on the lowest binding energy value [60]. The protein–ligand interaction conformation map is presented in Pymol. To ensure the accuracy and reliability of the docking program utilized in this study, we adopted and modified the method proposed by Chamata et al. [49]. The ligand Lisinopril was extracted from the protein database for human ACE (PDB: 1O86, resolution 2Å). Initially, the extracted ligand Lisinopril was redocked into the receptor protein. Upon completion of the docking process, we compared the generated docking conformations with the actual ligand structure within the protein using PyMOL 3.1 (Schrödinger, New Work, NY, USA). Subsequently, we calculated the RMSD value between the docked ligand and its corresponding structure in the protein. The success of the docking process depends on whether the RMSD value between the true and highest-scoring docking conformations during the docking process is within the 2 Å grid spacing [49,61].

### 4.6. Spectral Measurement

#### 4.6.1. UV Spectral Measurement

The UV spectra were determined by a UV-2600 spectrophotometer (Shimadzu, Kyoto, Japan). Different concentrations of the ACE-inhibitory peptides LLY and ACE (0.3 μmol·L^−1^, 0.25 U∙mg^−1^) were dissolved in 50 mmol·L^−1^ HEPES buffer (0.3 mol·L^−1^ NaCl, pH 7.0), and the buffer without ACE was corrected with a blank corresponding to the same concentration of LLY. After mixing and incubating at 25 °C for 10 min, the UV spectra were scanned in the 200–350 nm wavelength range.

#### 4.6.2. Fluorescence Spectrum Measurements

Fluorescence was measured with the F-7000 fluorescence spectrophotometer (Hitachi, Japan). Different concentrations of the ACE-inhibitory peptides LLY and ACE (0.3 μmol·L^−1^, 0.25 U∙mg^−1^) were dissolved in a 50 mmol·L^−1^ HEPES buffer (0.3 mol·L^−1^ NaCl, pH 7.0). The LLY solution was mixed with ACE solution and reacted in a 25 °C water bath shaker. After incubation for 10 min, the fluorescence spectra and 3D fluorescence spectra were respectively measured. For the fluorescence spectrum test, the excitation wavelength is fixed at 280 nm, and the fluorescence spectrum is scanned within 310–500 nm. The slit width for both incident and emitted light is set to 5.0 nm, with a scanning speed of 1200 nm∙min^−1^. For the 3D fluorescence spectrum test, the integration time is set to 0.1 s, with an excitation wavelength scanning range of 200–350 nm and an emission wavelength scanning range of 280–450 nm. The sampling interval for excitation and emission wavelengths is set at 5 nm, while the slit width remains at 5.0 nm and the integration time at 300 ms.

#### 4.6.3. CD Spectroscopic Measurements

CD spectral data were obtained from a MOS 450 CD spectrometer (Bio-Logic, Seyssinet-Pariset, EU, France) in rectangular quartz cuvettes of 1 mm path length. The GACE inhibitor peptide LLY (0.2 mmol·L^−1^) and ACE (0.5 μmol·L^−1^, 0.25 U∙mg^−1^) were dissolved in 50 mmol·L^−1^ HEPES buffer (0.3 mol·L^−1^ NaCl, pH 7.0). The LLY and ACE solutions were mixed and incubated at 25 °C for 10 min. Scanning was performed in the wavelength range of 190–250 nm with a scanning speed of 100 nm∙s^−1^ and a response time of 1 s. The background spectrum of the LLY solution was subtracted from that of the LLY−ACE complex.

### 4.7. AFM Measurement

AFM measurement was made with a Multimode 8 scanning probe microscope (Bruker, Beijing, China). The mica sheet without cracks is glued to the circular magnetic sheet; the sample is evenly dispersed and can be viewed on the AFM by natural air drying. Subsequently, it was observed using an AFM with the Scan Asyst Mode selected and a scanning range of 10 μm. Data processing and analysis were conducted using NanoScope Analysis 1.5 software (Bruker, Beijing, China).

### 4.8. Statistical Analysis

All of the experiments were performed in triplicate, and the mean values with standard deviation were reported. All data were analyzed by one-way ANOVA using SPSS17.0 software (Chicago, IL, USA). The linear correlation coefficient was calculated using Origin version 2018. A value of *p* < 0.05 was considered statistically significant.

## 5. Conclusions

In this study, the casein-derived ACE-inhibitory peptide LLY exhibits remarkable ACE inhibition efficacy, with an IC_50_ value of 44.16 ± 2.45 μmol·L^−1^, and demonstrates favorable stability properties. Therefore, we conducted a comprehensive study on the inhibition mechanism and stability of LLY and elucidated its interaction with ACE through multiple approaches. The Lineweaver–Burk diagram and the molecular docking analysis revealed that LLY can form seven hydrogen bonds with ACE, supporting its non-competitive inhibition property. Additionally, the interaction between LLY and ACE was investigated using UV spectroscopy, fluorescence spectroscopy, CD, and AFM. The results indicated that the addition of LLY altered the spatial conformation of ACE and formed a new complex with it, significantly impacting the secondary structure of ACE. Overall, these findings suggest that LLY possesses potent ACE-inhibiting activity and holds potential for application in hypertension treatment. However, further research is needed to elucidate the mechanism underlying the antihypertensive action of LLY in vivo.

## 6. Future and Prospect

The main object of study in this paper is the natural food-source peptide LLY, derived from casein, which is abundant in resources and readily available. Research indicates that the inhibitory peptide LLY shows promising research prospects as an antihypertensive component. In addition, this study has some limitations. The research on the novel ACE-inhibitory peptide LLY is still in the early stage, and further studies need to confirm its antihypertensive effect in vivo. Relevant animal experiments will be conducted to verify its efficacy and safety performance in vivo to evaluate the potential and application value of this new natural ACE inhibitor peptide more fully. This will help to provide a more reliable scientific basis for the further development and clinical application of this inhibitory peptide.

## Figures and Tables

**Table 1:** The 3D fluorescence spectral parameters of LLY and ACE.

System	Peak No	Peak Position [λ_ex_/λ_em_(nm/nm)]	Intensity
ACE	I	280/280	1029
	II	275/305	43.49
ACE + LLY	I	290/290	734.5
	II	275/305	2368

## Data Availability

The data presented in this study are available from the corresponding authors upon reasonable request.
